# The Control of V.A.D. Hospitals

**Published:** 1918-06-29

**Authors:** 


### THE CONTROL OF V.A.D. HOSPITALS.

### NEW ARMY COUNCIL INSTRUCTION.

To define more clearly the functions of the V.A.D. County Directors, an Army Council Instruction has been issued, of which the main provisions are :

The county director is the official head of the voluntary aid organisation in his county, and is responsible for all V.A.D. *personnel* engaged on transport work or in the hospitals under his jurisdiction.

He should also be recognised as an authorised honorary official of the military command. His duties are to satisfy himself of internal economy, numbers of staff engaged, and conditions under which members posted by him work, in all hospitals drawing capitation grants (except permanent civil hospitals and hospitals administered by boards of guardians or asylum committees) to which he posts V.A.D.s, for which he signs requisitions for stores, petrol, etc., or which apply to him for other assistance.

The military medical authorities should give the county director any information that may be required for carrying on the duties of the office, and will support the authority of the county director whenever necessary. The county director should establish close touch with the officers commanding the central hospitals to which the auxiliary hospitals are affiliated and with the A.D.M.S. of the district in which they are located, and should bring to the notice of these officers any irregularity which may be observed.

That more uniformity may be established in the finances and accounts of auxiliary hospitals, the authorities of all auxiliary hospitals should consult the county director on financial matters whenever necessary. It is the duty of the military authorities to ascertain that the patients are adequately fed and treated, and that they are not kept unduly long as inmates, for which purpose the military authorities will inspect all auxiliary hospitals at intervals.

